# Supplementary material for: Microbial dysbiosis reflects disease resistance in diverse coral species
Source: Commun Biol. 2021 Jun 3;4:679. doi: 10.1038/s42003-021-02163-5 (PMC8175568; doi:10.1038/s42003-021-02163-5)
Supplement: Supplementary file 2 — Supplemental Material [file 42003_2021_2163_MOESM2_ESM.pdf]

## Phenotype Supplemental

**Supplemental Table 1.** P-values from a pairwise Fisher's exact test of species disease prevalence. P-values not adjusted.

| | *O. annularis* | *P. astreoides* | *M. cavernosa* | *O. faveolata* | *C. natans* | *P. porites* |
|---|---|---|---|---|---|---|
| ***P. astreoides*** | 0.2063 | - | - | - | - | - |
| ***M. cavernosa*** | 0.0476 | 1.0000 | - | - | - | - |
| ***O. faveolata*** | 0.4444 | 0.0476 | 0.0079 | - | - | - |
| ***C. natans*** | 1.0000 | 0.2063 | 0.0476 | 1.0000 | - | - |
| ***P. porites*** | 0.2063 | 1.0000 | 1.0000 | 0.0476 | 0.2063 | - |
| ***S. siderastrea*** | 1.0000 | 0.5238 | 0.1667 | 0.4444 | 1.0000 | 0.5238 |

**Supplemental Table 2.** "Time" is hours of disease exposure before that fragment showed disease signs. "N.risk" number of fragments exposed to white plague during that time point which includes the fragment that became infected at that time point. "N.event" is the number of fragments that became infected at that time point. "Surv" is the proportion of fragments that have not contracted the disease at that time point.

| Species | time | n.risk | n.event | surv | std.err | lower 95% CI | Upper 95% CI |
|---|---|---|---|---|---|---|---|
| ***Orbicella faveolata*** | 116 | 5 | 1 | 0.8 | 0.179 | 0.5161 | 1 |
| | 116 | 4 | 2 | 0.4 | 0.219 | 0.1367 | 1 |
| | 119 | 2 | 1 | 0.2 | 0.179 | 0.0346 | 1 |
| | 136 | 1 | 1 | 0 | NaN | NA | NA |
| ***Orbicella annularis*** | 116 | 4 | 1 | 0.75 | 0.217 | 0.4259 | 1 |
| | 116 | 3 | 1 | 0.5 | 0.25 | 0.1877 | 1 |
| | 124 | 2 | 1 | 0.25 | 0.217 | 0.0458 | 1 |
| ***Colpophyllia natans*** | 39.9 | 5 | 1 | 0.8 | 0.179 | 0.5161 | 1 |
| | 88 | 4 | 1 | 0.6 | 0.219 | 0.2933 | 1 |
| | 112 | 3 | 1 | 0.4 | 0.219 | 0.1367 | 1 |
| | 124 | 2 | 1 | 0.2 | 0.179 | 0.0346 | 1 |
| ***Siderastrea siderea*** | 73.2 | 5 | 1 | 0.8 | 0.179 | 0.516 | 1 |
| | 89 | 4 | 1 | 0.6 | 0.219 | 0.293 | 1 |
| | 89.2 | 3 | 1 | 0.4 | 0.219 | 0.137 | 1 |
| ***Porites astreoides*** | 114 | 5 | 1 | 0.8 | 0.179 | 0.516 | 1 |
| ***Porites porites*** | 117 | 5 | 1 | 0.8 | 0.179 | 0.516 | 1 |
| ***Montastraea cavernosa*** | NA | NA | NA | NA | NA | NA | NA |

**Supplemental Table 3.** The number of coral fragments of each species that were assigned to one of three treatment outcomes. "C" represents control treatment. "DE" represents the disease-exposed treatment outcome and "DI" represents the disease-infected treatment outcome.

| | *O. faveolata* | *O. annularis* | *C. natans* | *S. siderea* | *P. porites* | *P. astreoides* | *M. cavernosa* | *Total* |
|---|---|---|---|---|---|---|---|---|
| ***C*** | **5** | **4** | **5** | **5** | **5** | **5** | **5** | **34** |
| ***DE*** | **0** | **1** | **1** | **2** | **4** | **4** | **5** | **17** |
| ***DI*** | **5** | **3** | **4** | **3** | **1** | **1** | **0** | **17** |

**Microbiome Supplemental**

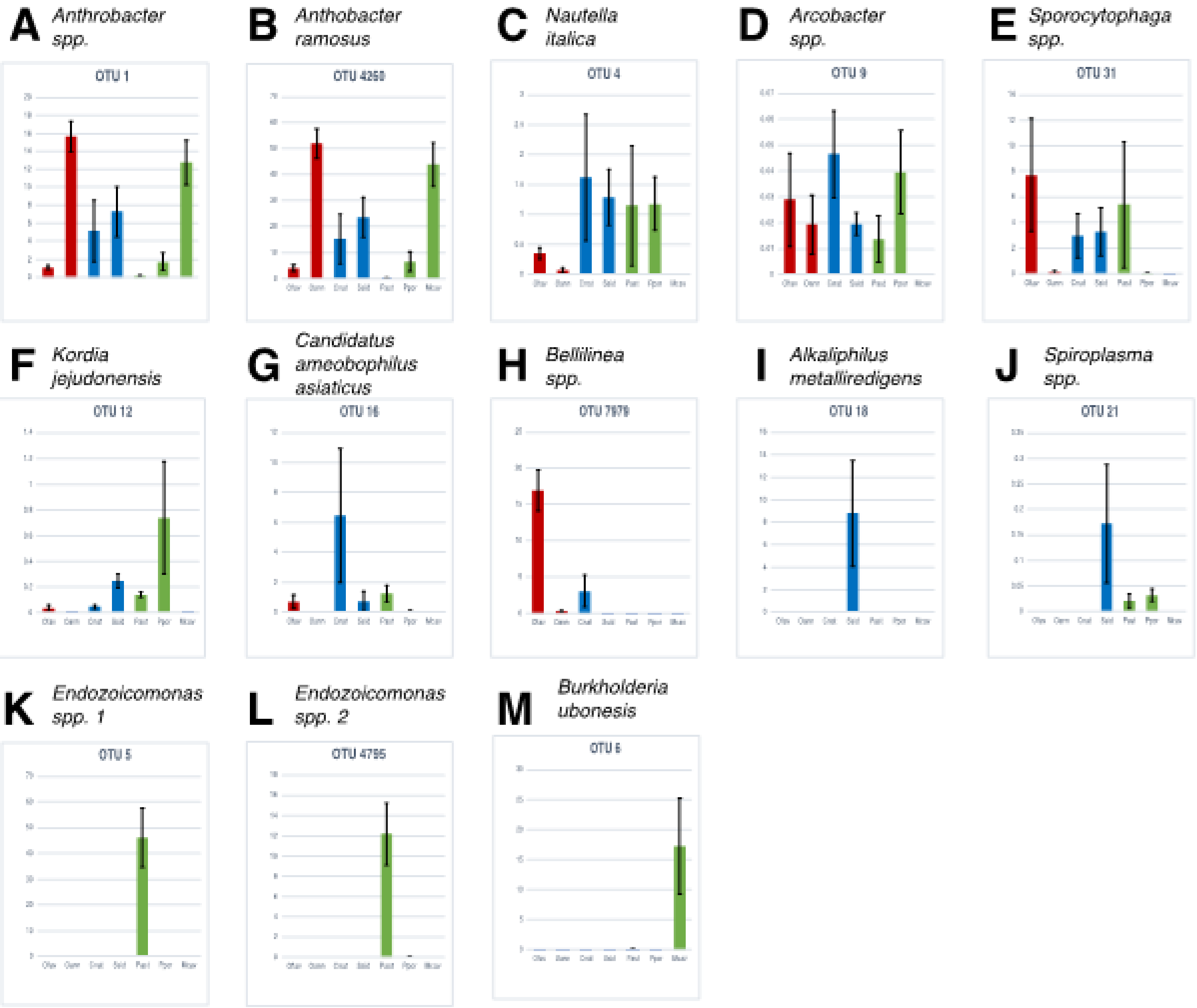


**Supplemental Figure 1**: Mean relative abundance (± SEM) of the bacterial OTUs that contributed the most to differences among coral species microbiomes for control coral fragments only based on SIMPER analysis. Red represents the highly susceptible species, blue for the intermediate, and green for the low susceptibility coral species.

**Supplemental Table 4**. Comparing lesion progression rate by species with a Tukey Post Hoc test.

| Pairs | Diff | Lwr | Upr | padj |
|---|---|---|---|---|
| *astreoides-annularis* | -0.0040 | -0.016266435 | 0.0082664353 | 0.8954887 |
| ***cavernosa-annularis*** | **-0.0110** | **-0.023266435** | **0.0012664353** | **0.0888854 .** |
| *faveolata-annularis* | -0.0016 | -0.009357975 | 0.0061579748 | 0.9870780 |
| ***natans-annularis*** | **-0.0085** | **-0.016613484** | **-0.0003865157** | **0.0382513 *** |
| *porites-annularis* | -0.0100 | -0.022266435 | 0.0022664353 | 0.1384242 |
| ***siderastrea-annularis*** | **-0.0090** | **-0.017673680** | **-0.0003263205** | **0.0404692 *** |
| *cavernosa-astreoides* | -0.0070 | -0.022023254 | 0.0080232537 | 0.6538771 |
| *faveolata-astreoides* | 0.0024 | -0.009236962 | 0.0140369622 | 0.9870780 |
| *natans-astreoides* | -0.0045 | -0.016376925 | 0.0073769249 | 0.8172609 |
| *porites-astreoides* | -0.0060 | -0.021023254 | 0.0090232537 | 0.7816972 |
| *siderastrea-astreoides* | -0.0050 | -0.017266435 | 0.0072664353 | 0.7667594 |
| *faveolata-cavernosa* | 0.0094 | -0.002236962 | 0.0210369622 | 0.1440396 |
| *natans-cavernosa* | 0.0025 | -0.009376925 | 0.0143769249 | 0.9856800 |
| *porites-cavernosa* | 0.0010 | -0.014023254 | 0.0160232537 | 0.9999771 |
| *siderastrea-cavernosa* | 0.0020 | -0.010266435 | 0.0142664353 | 0.9962160 |
| ***natans-faveolata*** | **-0.0069** | **-0.014026155** | **0.0002261549** | **0.0597342 .** |
| *porites-faveolata* | -0.0084 | -0.020036962 | 0.0032369622 | 0.2246988 |
| ***siderastrea-faveolata*** | **-0.0074** | **-0.015157975** | **0.0003579748** | **0.0647426 .** |
| *porites-natans* | -0.0015 | -0.013376925 | 0.0103769249 | 0.9990732 |
| *siderastrea-natans* | -0.0005 | -0.008613484 | 0.0076134843 | 0.9999855 |
| *siderastrea-porites* | 0.0010 | -0.011266435 | 0.0132664353 | 0.9999249 |

**Supplemental Table 5**. Pairwise permanova results from the pairwise.adonis function comparing treatment outcome.

| Pairs | F | R2 | p.value | p.adjusted |
|---|---|---|---|---|
| *Control vs Exposed* | 1.81 | 0.035 | 0.076 | 0.228 |
| *Control vs Infected* | 2.08 | 0.04 | 0.045 | 0.135 |
| *Exposed vs Infected* | 2.03 | 0.05 | 0.055 | 0.165 |

**Supplemental Table 6.** The required percent homology of sequences to reference sequence database to annotate OTUs at each classification level.

| Identity to reference sequence | Identity Designation |
|---|---|
| > 97% | Species |
| Between 97% and 95% | (unclassified Genus) |
| Between 95% and 90% | (unclassified Family) |
| Between 90% and 85% | (unclassified order) |
| Between 85% and 80% | (unclassified class) |
| Between 80% and 77% | (unclassified phylum) |
| < 77% | (unknown) |
