# Supplementary material for: Microbial dysbiosis reflects disease resistance in diverse coral species
Source: Commun Biol. 2021 Jun 3;4:679. doi: 10.1038/s42003-021-02163-5 (PMC8175568; doi:10.1038/s42003-021-02163-5)
Supplement: Supplementary file 3 — Reporting Summary [file 42003_2021_2163_MOESM3_ESM.pdf]

nature research

Corresponding author(s): Dr. Marilyn Brandt

Last updated by author(s): Mar 26, 2021

Reporting Summary

Nature Research wishes to improve the reproducibility of the work that we publish. This form provides structure for consistency and transparency in reporting. For further information on Nature Research policies, see our Editorial Policies and the Editorial Policy Checklist.

Statistics

For all statistical analyses, confirm that the following items are present in the figure legend, table legend, main text, or Methods section.

n/a	Confirmed	
☐	☒	The exact sample size (n) for each experimental group/condition, given as a discrete number and unit of measurement
☐	☒	A statement on whether measurements were taken from distinct samples or whether the same sample was measured repeatedly
☐	☒	The statistical test(s) used AND whether they are one- or two-sided<br>*Only common tests should be described solely by name; describe more complex techniques in the Methods section.*
☐	☒	A description of all covariates tested
☐	☒	A description of any assumptions or corrections, such as tests of normality and adjustment for multiple comparisons
☐	☒	A full description of the statistical parameters including central tendency (e.g. means) or other basic estimates (e.g. regression coefficient) AND variation (e.g. standard deviation) or associated estimates of uncertainty (e.g. confidence intervals)
☐	☒	For null hypothesis testing, the test statistic (e.g. F, t, r) with confidence intervals, effect sizes, degrees of freedom and P value noted<br>*Give P values as exact values whenever suitable.*
☐	☒	For Bayesian analysis, information on the choice of priors and Markov chain Monte Carlo settings
☐	☒	For hierarchical and complex designs, identification of the appropriate level for tests and full reporting of outcomes
☐	☒	Estimates of effect sizes (e.g. Cohen's d, Pearson's r), indicating how they were calculated

Our web collection on statistics for biologists contains articles on many of the points above.

Software and code

Policy information about availability of computer code

Data collection	ImageJ was used to measure lesion progression rates from photographic data.
Data analysis	Original code for analysis was written by authors.

For manuscripts utilizing custom algorithms or software that are central to the research but not yet described in published literature, software must be made available to editors and reviewers. We strongly encourage code deposition in a community repository (e.g. GitHub). See the Nature Research guidelines for submitting code & software for further information.

Data

Policy information about availability of data

All manuscripts must include a data availability statement. This statement should provide the following information, where applicable:

- Accession codes, unique identifiers, or web links for publicly available datasets
- A list of figures that have associated raw data
- A description of any restrictions on data availability

All data has been submitted for BCO-DMO with 16s rRNA data archived on NCBI.
NCBI: https://submit.ncbi.nlm.nih.gov/subs/sra/SUB8107847/overview
BCO-DMO Project Page: https://www.bco-dmo.org/project/727496

Field-specific reporting

Please select the one below that is the best fit for your research. If you are not sure, read the appropriate sections before making your selection.

☐ Life sciences ☐ Behavioural & social sciences ☒ Ecological, evolutionary & environmental sciences

For a reference copy of the document with all sections, see nature.com/documents/nr-reporting-summary-flat.pdf

Ecological, evolutionary & environmental sciences study design

All studies must disclose on these points even when the disclosure is negative.

Study description	Disease incidence and lesion progression rates were evaluated over the course of a seven-day exposure and were used as indicators of susceptibility among seven coral species tested. The microbiomes of corals was sampled after lesion appearance or at the end of the experiment if no disease signs appeared. A spectrum of disease susceptibility was observed among the seven coral species, which corresponded microbial dysbiosis to phenotypic responses.
Research sample	Five parental colonies from each of seven Caribbean coral species, Orbicella faveolata, Colpophyllia natans, Siderastrea siderea, Porites astreoides, Porites porites, and Montastraea cavernosa, were collected from Brewers Bay (18.34403, -64.98435), St Thomas, U.S. Virgin Islands on 13 June 2017
Sampling strategy	When a disease lesion appeared on a disease-exposed coral that was previously healthy it was monitored until 30% tissue loss. If the lesion enlarged over this time period, the coral and its paired control fragment were photographed, removed, flash frozen and stored at -80˚C until further 16S rRNA analysis.
Data collection	Photographic data was recorded twice a day by Dr. Marilyn Brandt. DNA extractions were collected by a DNeasy Powersoil Extraction Kit by Nick MacKnight and Kathryn Cobleigh for 16s rRNA sequencing of the v4 region on a MiSeq.
Timing and spatial scale	When a disease lesion appeared on a disease-exposed coral that was previously healthy it was monitored until 30% tissue loss. If the lesion enlarged over this time period, the coral and its paired control fragment were photographed, removed, flash frozen and stored at -80˚C until further 16S rRNA analysis.
Data exclusions	No exclusions.
Reproducibility	Experimental methods are originally detailed in Williams et al. 2020 (M Brandt is corresponding author)
Randomization	Containers received water changes daily and their locations were also randomized each day over the course of the 7-day experimental period. Each treatment container consisted of a randomly assigned healthy fragment of each of the seven tested species that were placed equal distances (approximately 7-8 cm) from a central diseased O. franksi fragment. Control containers were identically arranged, except that healthy O. franksi were used as the central corals (Supplemental Figure 1).
Blinding	Not relevant.

Did the study involve field work? ☒ Yes ☐ No

Field work, collection and transport

Field conditions	Experimental corals were collected from the field by divers on SCUBA using hammers and chisels on 13 June 2017. No adverse conditions were noted at the time of collection and all corals appeared healthy.
Location	Brewers Bay (18.34403, -64.98435), St. Thomas, US Virgin Islands
Access & import/export	All coral collections were performed under the auspices of the US Virgin Islands Division of Fish and Wildlife Research and Export Permit #CZM17010T.
Disturbance	Experimental corals were collected by scientific divers on SCUBA using hammers and chisels and under the guidance of a territorial research and export permit. For smaller and highly abundant species (e.g., Porites astreoides) whole colonies were taken. For larger coral species, only sub-samples were taken and areas where sampled were covered with modeling clay to accelerate healing.

Reporting for specific materials, systems and methods

We require information from authors about some types of materials, experimental systems and methods used in many studies. Here, indicate whether each material, system or method listed is relevant to your study. If you are not sure if a list item applies to your research, read the appropriate section before selecting a response.

Materials & experimental systems

n/a	Involved in the study
x	Antibodies
x	Eukaryotic cell lines
x	Palaeontology and archaeology
	x Animals and other organisms
x	Human research participants
x	Clinical data
x	Dual use research of concern

Methods

n/a	Involved in the study
x	ChIP-seq
x	Flow cytometry
x	MRI-based neuroimaging

Animals and other organisms

Policy information about studies involving animals; ARRIVE guidelines recommended for reporting animal research

Laboratory animals	*For laboratory animals, report species, strain, sex and age OR state that the study did not involve laboratory animals.*
Wild animals	Orbicella faveolata, Orbicella annularis, Orbicella franksi, Colpophyllia natans, Siderastrea siderea, Porites astreoides, Porites porites, and Montastraea cavernosa.
Field-collected samples	Experimental organisms were collected from the field, transported in coolers filled with seawater and then kept in shaded outdooor flow-through seawater tanks that are part of the MacLean Marine Science Center at the University of the Virgin Islands. Seawater was pumped from Brewers Bay into a sediment settling cistern and then gravity fed to a second sediment settling tank. From there seawater was pumped through a chiller to keep temperatures consistent with ambient reef levels and then also through a UV filter.
Ethics oversight	No ethical approval was required.

Note that full information on the approval of the study protocol must also be provided in the manuscript.
